# Standardizing Sperm Vitality and Acrosomal Integrity Assays for the Conservation of *Apis mellifera* and *Scaptotrigona mexicana*

**DOI:** 10.3390/insects17101080

**Published:** 2026-10-19

**Authors:** Mario Blanco-Camarillo, Natalia Real-Luna, Ma. Teresa Santillán-Galicia, Horacio Álvarez-Gallardo, Jesús Romero-Nápoles, Marcelino Martínez-Núñez, Víctor Zúñiga-Mayo, Juan Antonio Pérez-Sato, David Urbán-Duarte

**Affiliations:** 1 Fitosanidad, Colegio de Postgraduados, Carretera Federal México–Texcoco km 36.5, Montecillo 56230, Mexico; marioc.s@hotmail.com (M.B.-C.); tgalicia@colpos.mx (M.T.S.-G.); jnapoles@colpos.mx (J.R.-N.); martinez.marcelino@colpos.mx (M.M.-N.); zuniga.victor@colpos.mx (V.Z.-M.); 2 Colegio de Postgraduados Campus Córdoba, Carretera Federal Córdoba–Veracruz km 348, Congregación Manuel León, Amatlán de los Reyes 94953, Mexico; nreal@colpos.mx (N.R.-L.); pantonio@colpos.mx (J.A.P.-S.); 3 Centro Nacional de Recursos Genéticos (CNRG-INIFAP), Blvd. de la Biodiversidad No. 400, Tepatitlán de Morelos 47600, Mexico; alvarez.horacio@inifap.gob.mx; 4 Secretaría de Ciencia, Humanidades, Tecnología e Innovación (SECIHTI), Mexico City 03940, Mexico

**Keywords:** drone semen, propidium iodide, FITC-PNA, semen extender, fluorescence microscopy

## Abstract

Reliable assessment of bee sperm quality is important for breeding programs, artificial insemination, and germplasm conservation. However, incubation conditions for combined fluorescence assessment remain poorly standardized across bee species. This study evaluated a sequential triple-staining protocol using Hoechst 33342, propidium iodide, and FITC-PNA in *Apis mellifera* and *Scaptotrigona mexicana*. Five incubation times (5, 10, 15, 20, and 30 min per fluorochrome) were tested. The shortest tested intervals allowing consistent fluorescence interpretation across all five microscopic fields in every biological replicate were 20 min per fluorochrome in *A. mellifera* and 30 min per fluorochrome in *S. mexicana*. Using these conditions, sperm vitality and acrosomal integrity were assessed by fluorescence microscopy after one week of storage at 22 °C in FEM7, saline solution (SS), or modified Kiev. Sperm vitality was determined by Hoechst 33342/PI live–dead classification, and localized green FITC-PNA fluorescence was the criterion for acrosomal damage. Binomial generalized linear mixed models were used to analyze the observed counts in relation to species, diluent, and their interaction. A significant species × diluent interaction was detected. Modified Kiev yielded the highest estimated sperm vitality in *A. mellifera*, without a significant difference from FEM7, whereas FEM7 yielded the highest estimated sperm vitality in *S. mexicana*. SS yielded the lowest estimated sperm vitality in both species. Acrosomal damage was detected exclusively in spermatozoa classified as dead. These results establish operational staining conditions for the combined assay and show that the relative performance of complete diluent formulations differed between species under the collection and storage conditions tested.

## Simple Summary

We evaluated a sequential staining procedure using Hoechst 33342, propidium iodide, and FITC-PNA in the honey bee (*Apis mellifera*) and the stingless bee (*Scaptotrigona mexicana*). Among the incubation times tested, all examined fields yielded evaluable fluorescence after 20 min per fluorochrome in *A. mellifera* and 30 min per fluorochrome in *S. mexicana*. We then compared sperm samples maintained in three diluents. The proportion of spermatozoa classified as live differed among diluents and between species. These results provide defined conditions for fluorescence-based sperm assessment and identify questions for further work on semen handling in both species.

## 1. Introduction

Bees are among the most important pollinators worldwide, playing a key role in ecosystem stability [1]. In addition, bees contribute directly to human livelihoods by providing hive products such as honey, wax and propolis, but more importantly they support rural economies through pollination services that sustain crop production and generate substantial economic value on a global scale [2]. Therefore, the sustainability of bee populations has direct ecological, agricultural, and economic implications.

Beyond their ecological and economic value, colony persistence in social bees depends on reproductive efficiency, as queens mate only briefly early in life and must rely on a finite sperm store in the spermatheca to fertilize eggs throughout their lifespan [3]. In honey bees (*Apis mellifera*), stored sperm in the spermatheca can remain viable and capable of fertilization for up to five years, although its quality gradually declines due to physiological and metabolic changes [4]. Because queens cannot replenish sperm after mating, lifetime reproductive output is determined by the small fraction of sperm successfully stored during mating flights and by the mechanisms that regulate its long-term use [5].

Consequently, sperm quality in drones is critical for colony performance, as sperm viability, particularly membrane integrity, directly influences successful storage within the spermatheca and is closely associated with subsequent fertilization capacity and the production of viable worker offspring [6]. Reduced sperm vitality in queens has been strongly associated with reduced reproductive performance and colony failure, with queens from healthy colonies showing much higher sperm viability (~85–92%) than those from failing colonies (~50–55% viability) [7]. These findings underscore the importance of developing reliable and standardized semen quality assessment methods, as current approaches in honey bees remain limited and often rely on single parameters, highlighting the need for multiparametric evaluations capable of accurately assessing sperm functionality and improving outcomes in breeding programs, artificial insemination, and genetic conservation strategies [8].

Stingless bees (Meliponini) are key pollinators in Mesoamerica, contributing directly to the pollination of wild and cultivated plants and enhancing crop yield and quality, while also holding ecological, economic, and cultural importance within traditional meliponiculture systems [9]. Despite their importance, reproductive biology and semen assessment in stingless bees such as *Scaptotrigona mexicana* remain poorly studied. Although studies have been conducted in some *Scaptotrigona* species [10], methodologies have largely been adapted from the honey bee, and no standardized protocols are currently available for *S. mexicana*, limiting comparative reproductive assessments and highlighting the need for species-specific optimization of semen evaluation protocols and reproductive parameters.

Several semen extenders have been developed to improve sperm handling, short-term storage, and cryopreservation in *A. mellifera*. Among these, Kiev-based formulations and FEM7 have shown favorable performance under different preservation conditions. However, their effectiveness has been evaluated almost exclusively in honey bees, and it remains unknown whether these extenders provide an equally suitable physicochemical environment for stingless bee spermatozoa. Given the physiological differences in reproductive biology between honey bees and Meliponini, comparative evaluation of these extenders is necessary before they can be applied to native bee species.

Fluorescent staining techniques are widely used to assess sperm viability across taxa. In honey bees, a supravital dual-staining method using bisbenzimide (Hoechst 33342) and propidium iodide (PI) has been applied to differentiate membrane-intact from membrane-compromised sperm cells [11]. Although *A. mellifera* drone semen has been included in multiparametric fluorescence analyses, acrosomal assessment using lectin-based probes such as peanut agglutinin (PNA) remains technically limited. This limitation is primarily due to the small size and reduced morphological differentiation of the sperm head, preventing robust validation in this species [12].

Moreover, fluorescence-based assays are highly sensitive to methodological factors including fixation protocols, staining procedures, and sample processing conditions. These factors can significantly alter the stability and interpretation of fluorescence signals, thereby influencing the accuracy of viability and acrosomal integrity estimates [13]. Although these methodological considerations have been partially addressed in other taxa, their systematic evaluation remains limited in honey bees and has not been studied in *S. mexicana*. Therefore, it is important to develop protocols specifically adapted to morphological and physiological traits of these species.

This study evaluated two aspects of fluorescence-based sperm assessment in *A. mellifera* and *S. mexicana*. First, we identified the shortest tested incubation time per fluorochrome that yielded consistently evaluable fields with a sequential Hoechst 33342, PI, and FITC-PNA protocol. We then used the selected staining conditions to compare sperm samples maintained in modified Kiev, FEM7, and saline solution. Fluorescence microscopy was used to visualize the staining signals, while the coupled Sperm Class Analyzer system provided image acquisition, automated sperm counting and live–dead classification, and analysis reports containing the counts and percentages used in the evaluations. Binomial generalized linear mixed models were used to analyze the resulting counts in relation to species, diluent, and their interaction.

## 2. Materials and Methods

### 2.1. Reagents and Media

All chemicals were purchased from Sigma–Aldrich (St. Louis, MO, USA). All media were prepared using ultrapure water under sterile conditions. Three semen extenders were evaluated in both bee species: modified Kiev [14], FEM7 [15], and saline solution (SS). The modified Kiev extender contained 82.21 mM sodium citrate, 24.87 mM sodium bicarbonate (NaHCO_3_), 5.34 mM potassium chloride (KCl), 16.7 mM glucose, 2% (*w*/*v*) bovine serum albumin (BSA), and 1 mL of Antibiotic Mixture 1 per 100 mL of extender. Antibiotic Mixture 1 consisted of gentamicin (50 mg/mL) and tylosin (200 mg/mL) mixed with nanopure water in a 5:2:3 (*v*/*v*/*v*) ratio. The pH of the Kiev-BSA extender was adjusted to 8.1, and its measured osmolality was approximately 385 mOsm kg^−1^. FEM7 consisted of 0.3029 g Tris, 0.4383 g NaCl, 0.5629 g KCl, 0.8558 g sucrose, 0.4504 g fructose, 0.0475 g EGTA, 0.0364 g EDTA, 250 µL of Antibiotic Mixture 1, and distilled water to a final volume of 50 mL. SS consisted of 0.275 g NaCl and 0.155 g KCl dissolved in distilled water to a final volume of 50 mL. Modified phosphate-buffered saline (PBS; 136.89 mM NaCl, 2.68 mM KCl, 8.09 mM Na_2_HPO_4_·7H_2_O, 1.47 mM KH_2_PO_4_, 0.90 mM CaCl_2_·2H_2_O, 0.49 mM MgSO_4_·7H_2_O, 5.55 mM glucose, and 0.33 mM sodium pyruvate) [16] was used for the preparation and dilution of the fluorochromes for fluorescence analysis.

### 2.2. Study Species and Sample Collection

**Figure 1:**
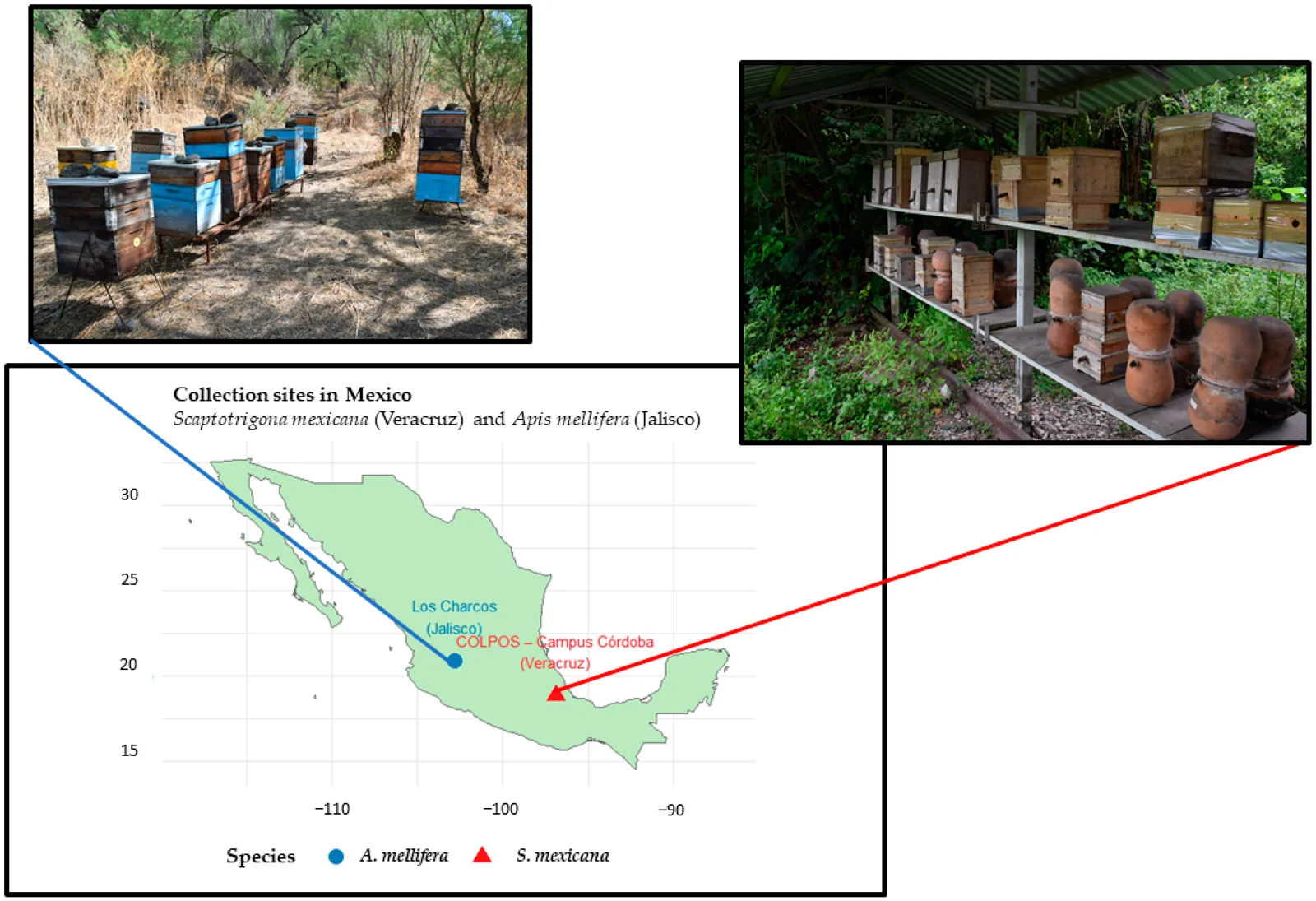
Geographic location of sampling sites for *Apis mellifera* and *Scaptotrigona mexicana*.

Adult drones of *A. mellifera* were collected in Los Charcos, Jalisco, Mexico (20.8866° N, −102.8086° W), during active flight periods at hive entrances using entomological nets. Drones of *S. mexicana* were obtained from colonies maintained at the Colegio de Postgraduados Campus Córdoba, Veracruz, Mexico (18.8560° N, −96.8601° W) (Figure 1), and were captured near the colony entrance during aggregation activity. All individuals were transported to the laboratory in ventilated containers and processed within one hour of collection to ensure sample integrity.

### 2.3. Semen Collection and Dilution

**Figure 2:**
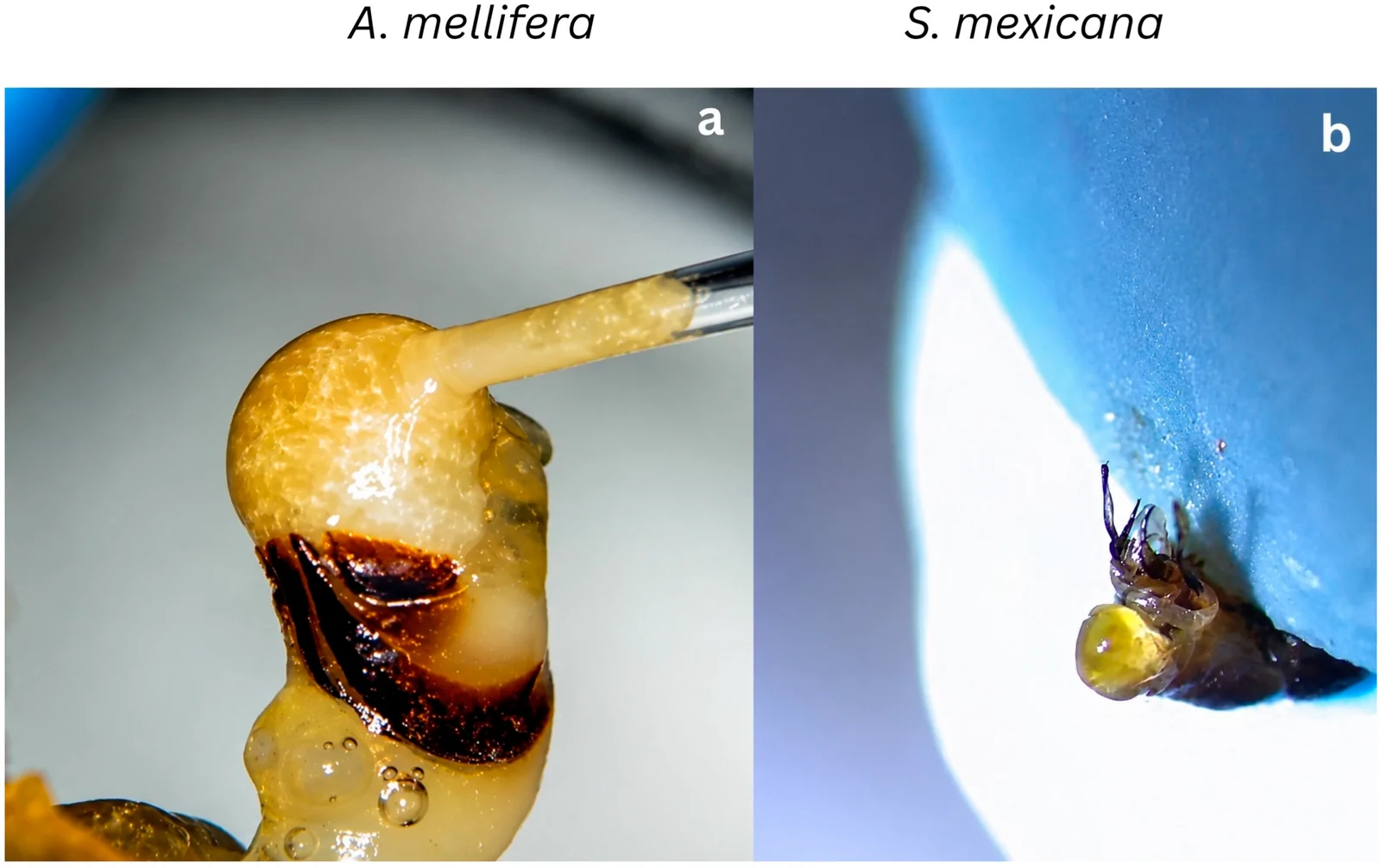
Semen collection procedures in *Apis mellifera* and *Scaptotrigona mexicana*. (**a**) Manual eversion of the endophallus in *A mellifera* by gentle abdominal pressure. (**b**) Extraction of seminal vesicles in *S. mexicana* following controlled thoracic and abdominal pressure.

In *A. mellifera*, semen was obtained by manual eversion of the endophallus induced by applying gentle pressure to the abdomen and thorax. For the diluent experiment, three independent pools were prepared, each containing ejaculated semen from 100 distinct drones, for a total of 300 drones. Each pool constituted one biological replicate and was subdivided into aliquots for evaluation in modified Kiev, FEM7, and SS. For each extender, 2 µL of pooled semen was diluted in 198 µL of medium, giving a 1:100 dilution. Each suspension was gently homogenized by pipetting (Figure 2a).

In *S. mexicana*, seminal vesicles were extracted by applying slight pressure to the thorax and abdomen and carefully removed using fine dissection forceps. Three biological replicates were evaluated per extender. Within each replicate, three seminal vesicles were assigned to each extender, with each vesicle individually transferred into 100 µL of modified Kiev, FEM7, or SS and gently disrupted by repeated pipetting to release the spermatozoa. Each resulting sperm suspension was maintained and evaluated separately; no pooling of seminal vesicles or sperm suspensions was performed. Thus, nine vesicles were evaluated per extender, giving a total of 27 vesicles across the three extenders and three biological replicates (Figure 2b).

Semen collection differed between species. Ejaculated semen was obtained from *A. mellifera*, whereas spermatozoa were released directly from seminal vesicles in *S. mexicana*. These procedures may result in different amounts and compositions of extracellular seminal material remaining in the sperm suspensions after dilution. Interactions between this material and the experimental extenders may therefore contribute to the observed differences in sperm vitality and acrosomal integrity. Extracellular seminal constituents were not quantified, so their individual contributions could not be determined. Because collection procedure and species were not varied independently, their respective contributions to between-species differences cannot be separated.

### 2.4. Fluorescent Staining Protocol

Sperm vitality and FITC-PNA staining patterns were evaluated using Hoechst 33342 (Sigma–Aldrich), propidium iodide (PI; Sigma–Aldrich), and FITC-conjugated peanut agglutinin (FITC-PNA; Sigma–Aldrich). For each sample, an 80 µL aliquot of sperm suspension diluted 1:100 in the corresponding semen extender was transferred to a sterile microcentrifuge tube. Staining was performed at room temperature (22–25 °C) with samples protected from light to limit photobleaching.

The fluorochromes were added sequentially. First, 8 µL of Hoechst 33342 (0.05 mg/mL in PBS) was added and incubated for 5, 10, 15, 20, or 30 min, depending on the treatment. Next, 2 µL of PI (0.5 mg/mL in PBS) was added and incubated for the same duration. Finally, 2 µL of FITC-PNA (200 µg/mL in PBS) was added and incubated for the same duration. Thus, the stated incubation time applied separately to each fluorochrome, and total staining time comprised three consecutive intervals of equal length. No washing steps were performed between fluorochrome additions or before microscopy. After the final incubation, 8 µL of the stained suspension was loaded into a Makler chamber for fluorescence evaluation.

### 2.5. Microscopy and Image Acquisition

Fluorescence observations were performed using a Nikon Eclipse E200 fluorescence microscope ((Nikon Corporation, Tokyo, Japan) equipped with UV-2A and B-2A filter sets. The coupled CASA system (Sperm Class Analyzer, version 6.6) was used for fluorescence image acquisition and automated sperm counting and live–dead classification according to the Hoechst 33342/PI fluorescence criteria. The system generated analysis reports containing the live–dead counts and corresponding percentages used in the evaluations.

Fluorescence evaluability was defined as the number of microscopic fields (0–5) in which spermatozoa exhibited consistent and detectable fluorescence signals following the sequential fluorochrome incubations.

In the diluent experiment, three biological replicates were evaluated per species and extender. For each individual sperm suspension, 100 spermatozoa were counted in each of five non-overlapping microscopic fields, giving 500 spermatozoa per suspension. In *A. mellifera*, one suspension per extender was evaluated from each independent semen pool. In *S. mexicana*, three individual vesicle-derived suspensions were evaluated per extender within each biological replicate, giving 1500 spermatozoa per replicate and extender.

Acrosomal integrity and sperm vitality were recorded separately in the same microscopic fields, using FITC-PNA and Hoechst 33342/PI, respectively.

### 2.6. Experimental Design

The study consisted of two sequential experimental phases. In the first phase, a completely randomized factorial design was used to optimize the fluorescence staining protocol. Sperm samples diluted in FEM7 extender were used for protocol standardization, with species (*A. mellifera* and *S. mexicana*) and incubation time (5, 10, 15, 20, and 30 min per fluorochrome) as fixed factors. Five biological replicates were analyzed for each species and incubation time. Incubation-time optimization was conducted as a separate experimental phase; the one-week storage period described below applied only to the subsequent diluent evaluation.

In the second phase, the incubation times selected in the first phase were applied to evaluate sperm vitality and acrosomal integrity in FEM7, modified Kiev, and SS after one week of storage at 22 °C. Three biological replicates were evaluated per species and extender. In *A. mellifera*, three independent semen pools were prepared, each obtained from 100 distinct drones, involving 300 drones in total. Each pool constituted one biological replicate and was subdivided into aliquots assigned to the three extenders. In *S. mexicana*, each biological replicate included three individually maintained seminal vesicles per extender. The nine vesicles within each replicate were processed separately, without pooling, giving nine vesicles per extender and 27 vesicles in total across the three biological replicates. Five microscopic fields were evaluated per sperm suspension, with 100 spermatozoa counted per field. The individual vesicle-derived suspensions in *S. mexicana* were nested within biological replicate and extender, and microscopic fields and spermatozoa were subsamples within each suspension.

### 2.7. Statistical Analysis

In the incubation-time experiment, fluorescence evaluability, expressed as the number of evaluable microscopic fields out of five, was analyzed using a generalized linear mixed model (GLMM) with a binomial distribution and logit link function. Species, incubation time, and their interaction were included as fixed effects, and biological replicate was included as a random effect.

The sampling structure differed between species. In *A. mellifera*, aliquots from each of three independent semen pools were evaluated in all three extenders, creating a split-sample structure. In *S. mexicana*, three individual vesicle-derived sperm suspensions were evaluated per extender within each of three biological replicates. These suspensions were processed and evaluated separately, without pooling, and were nested within biological replicate and extender. Five microscopic fields were examined per suspension, with 100 spermatozoa counted per field. Microscopic fields and individual spermatozoa were subsamples within each suspension.

Model-estimated proportions and their corresponding 95% confidence intervals were obtained from the fitted models. Pairwise comparisons among diluents within each species were performed using Tukey-adjusted contrasts, with a significance level of α = 0.05. All analyses were conducted in R version 4.5.3 using the packages *lme4* and *emmeans*.

## 3. Results

### 3.1. Fluorescence Stability and Optimal Incubation Time

**Figure 3:**
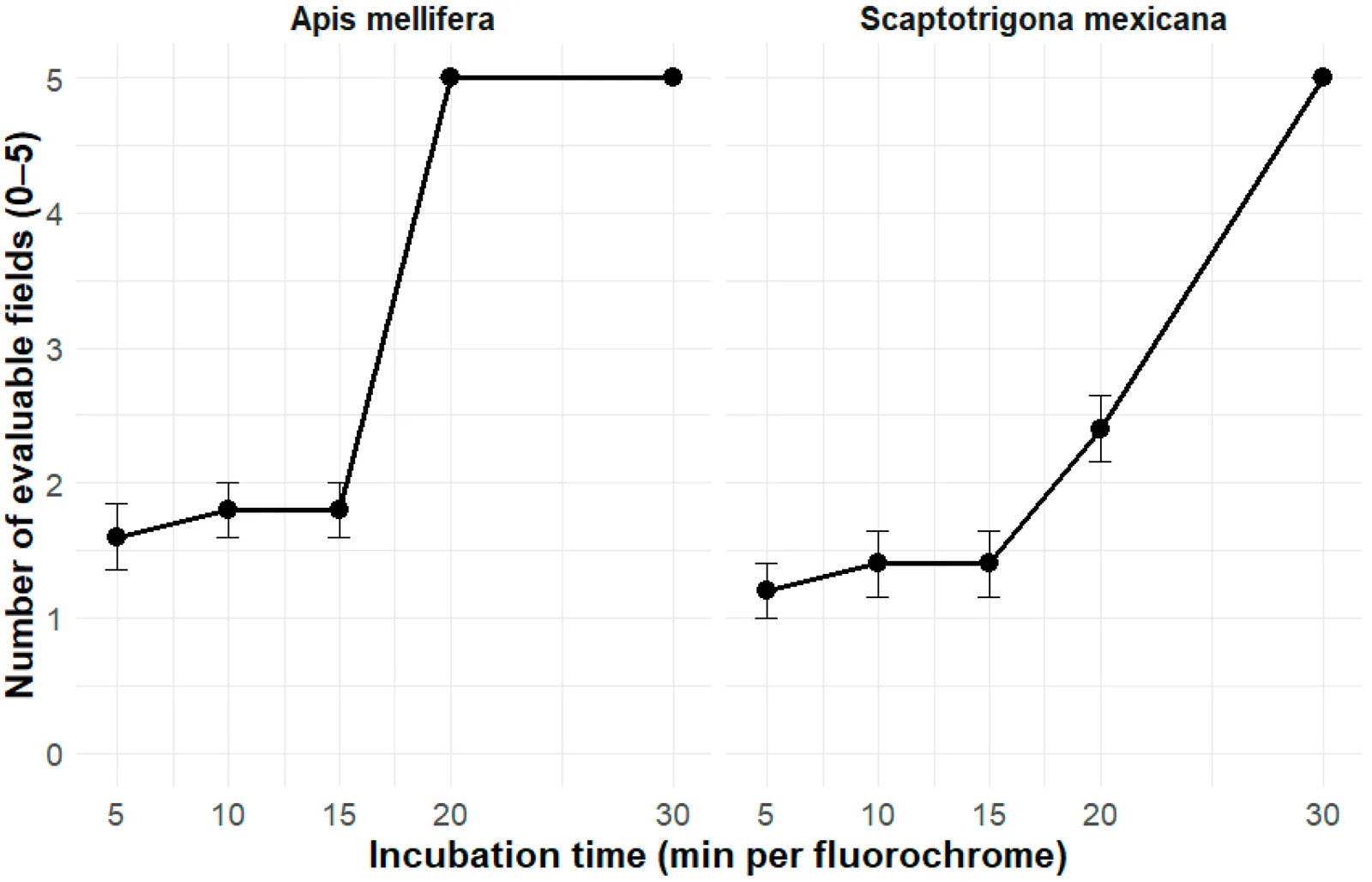
Number of evaluable microscopic fields at each fluorochrome incubation time in *A. mellifera* and *S. mexicana*. Five biological replicates were evaluated per species and time, with five fields examined per replicate.

The incubation-time experiment identified the shortest tested interval that allowed for consistent interpretation of the fluorescence signals across all five microscopic fields in every biological replicate. These intervals were 20 min per fluorochrome in *A. mellifera* and 30 min per fluorochrome in *S. mexicana* (Figure 3) and were subsequently used for sperm vitality and acrosomal integrity assessment in the diluent experiment.

These results defined the species-specific incubation times required for reliable fluorescence detection, which were used in all subsequent analyses.

### 3.2. Qualitative Assessment of Sperm Vitality

**Figure 4:**
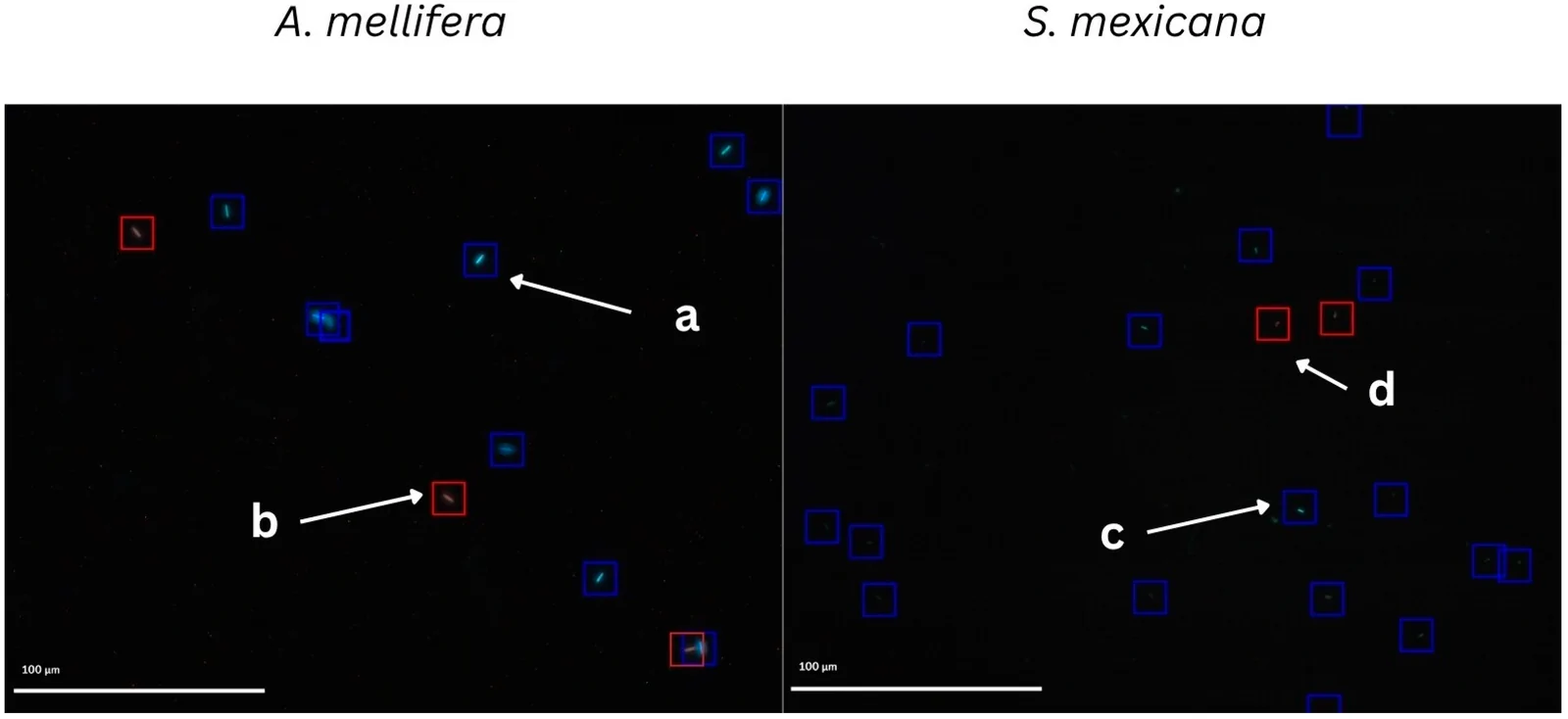
Fluorescence-based discrimination of sperm vitality in *Apis mellifera* and *Scaptotrigona mexicana*. Representative fluorescence micrographs showing viable and non-viable spermatozoa under optimized staining conditions. Blue nuclear fluorescence corresponds to Hoechst 33342 staining, whereas red fluorescence corresponds to propidium iodide (PI) staining. In *A. mellifera*, (a) viable spermatozoa (Hoechst-positive/PI-negative) and (b) non-viable spermatozoa (PI-positive). In *S. mexicana*, (c) viable spermatozoa (Hoechst-positive/PI-negative) and (d) non-viable spermatozoa (PI-positive).

Representative fluorescence micrographs obtained under the selected staining conditions showed the fluorescence patterns used to distinguish live and dead spermatozoa in both bee species (Figure 4). Spermatozoa exhibiting blue nuclear fluorescence without PI staining (Hoechst-positive/PI-negative) were classified as live, whereas PI-positive spermatozoa were classified as dead. This classification was based on plasma membrane integrity.

### 3.3. Qualitative Assessment of Acrosomal Integrity

**Figure 5:**
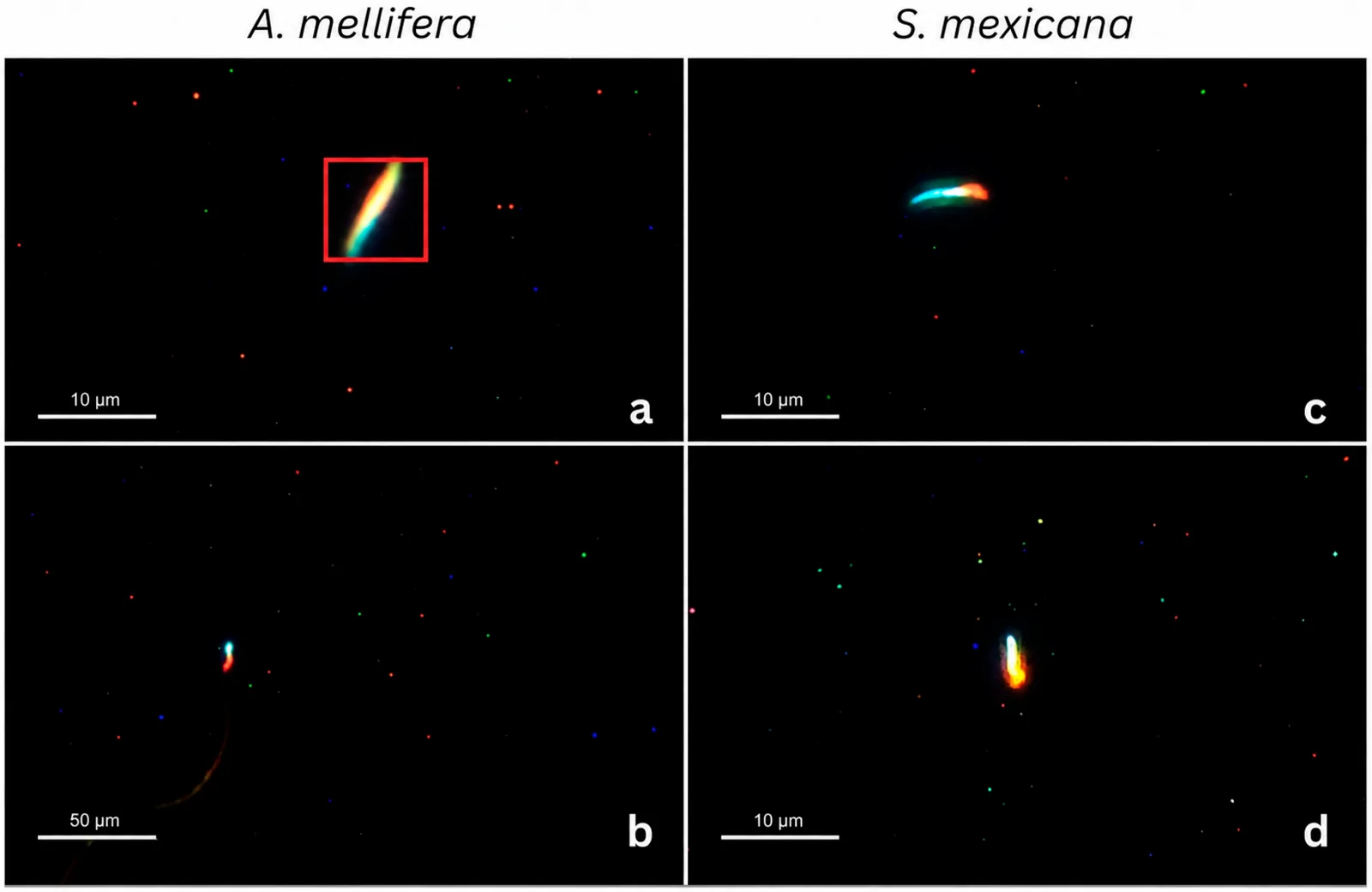
Representative fluorescence micrographs of spermatozoa classified as having damaged acrosomes by FITC-PNA staining in *Apis mellifera* and *Scaptotrigona mexicana*. Panels (**a**,**b**) show *A. mellifera*, and panels (**c**,**d**) show *S. mexicana*. Green fluorescence corresponds to FITC-PNA staining. Localized green fluorescence in the presumed acrosomal region was the operational criterion used to classify spermatozoa as having damaged acrosomes. Only spermatozoa classified as having damaged acrosomes are shown.

FITC-PNA staining revealed localized green fluorescence in the acrosomal region of spermatozoa in both species (Figure 5). According to the classification criteria described in Section 2.5, spermatozoa exhibiting this fluorescence were classified as having damaged acrosomes, whereas those without detectable green fluorescence in this region were classified as having intact acrosomes.

### 3.4. Effect of Diluents on Sperm Vitality and Acrosomal Integrity

Using the species-specific optimized incubation times (20 min per fluorochrome for *A. mellifera* and 30 min per fluorochrome for *S. mexicana*), the effects of three semen extenders (FEM7, modified Kiev, and SS) on sperm vitality and acrosomal integrity were evaluated using binomial generalized linear mixed models (GLMM). Significant effects of species, extender, and their interaction were detected, indicating that extender performance differed between species (all *p* < 0.001).

**Figure 6:**
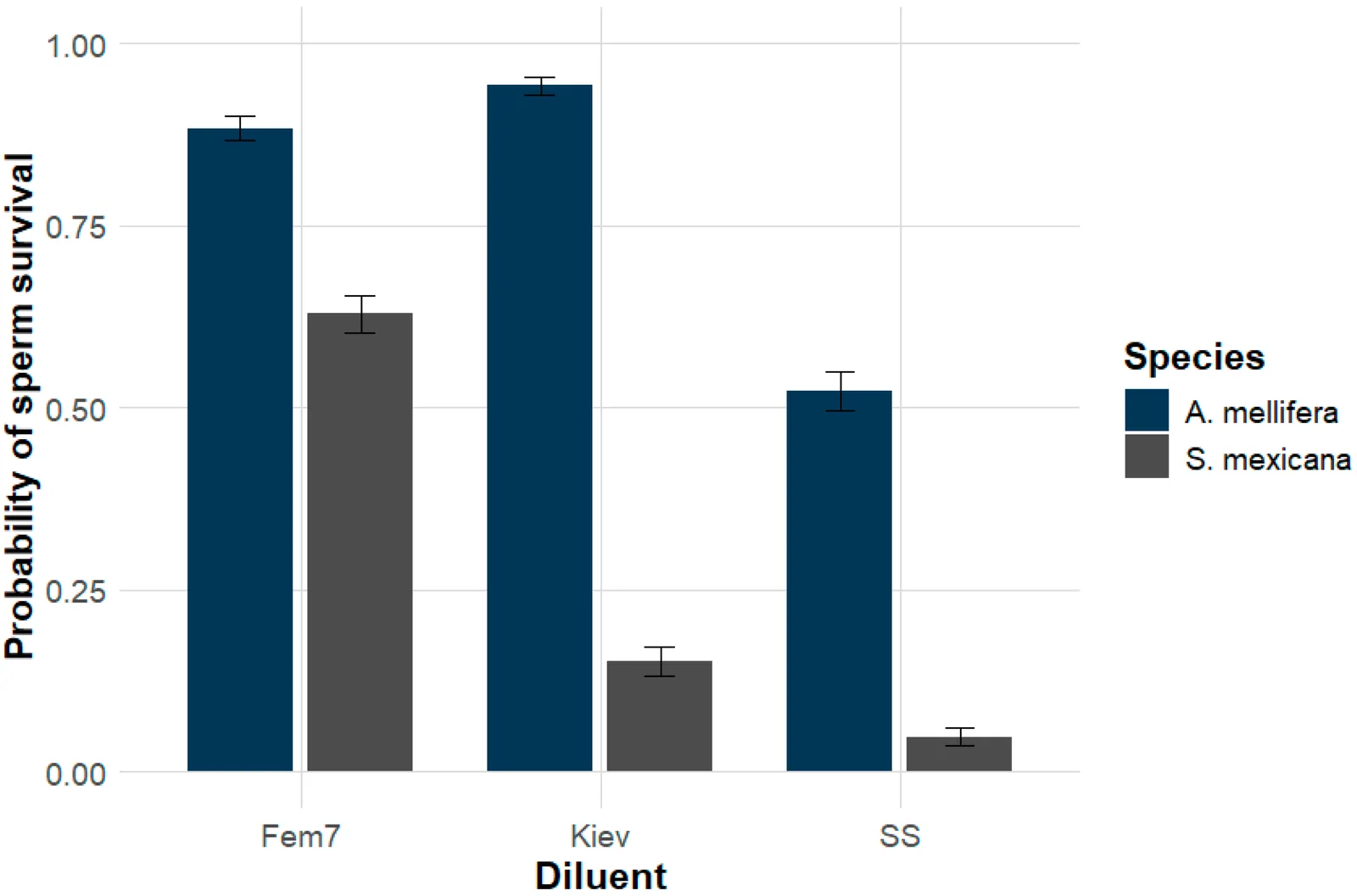
Estimated proportion of live spermatozoa in *A. mellifera* and *S. mexicana* by semen extender, derived from a binomial GLMM fitted to observed Hoechst 33342/PI live–dead counts.

Tukey-adjusted pairwise comparisons identified significant differences between specific extender pairs within each species (Figure 6). In *A. mellifera*, modified Kiev yielded the highest predicted sperm vitality (approximately 95%), followed by FEM7 (88%), whereas SS yielded significantly lower vitality (approximately 53%; *p* < 0.05). Modified Kiev and FEM7 did not differ significantly, and both yielded significantly higher vitality than SS. In *S. mexicana*, FEM7 yielded the highest predicted sperm vitality (approximately 63%), whereas modified Kiev (15%) and SS (5%) yielded significantly lower values (*p* < 0.05). No significant difference was detected between modified Kiev and SS.

The GLMM detected significant effects of species (F = 30.80, *p* < 0.001), extender (F = 86.38, *p* < 0.001), and the species × extender interaction (F = 50.11, *p* < 0.001) on acrosomal damage. Within each species, FEM7 yielded the lowest model-estimated proportion of spermatozoa with damaged acrosomes, whereas SS yielded higher values. In *S. mexicana*, modified Kiev also yielded a higher estimated proportion than FEM7. Across all treatments, acrosomal damage was detected exclusively in spermatozoa classified as dead by PI staining. Between-species differences must be interpreted in the context of the different collection procedures, because species and collection method were not evaluated independently.

## 4. Discussion

Among the incubation times tested, complete field evaluability was first observed at 20 min per fluorochrome in *A. mellifera* and 30 min per fluorochrome in *S. mexicana*. In the subsequent diluent experiment, sperm vitality differed among formulations, and the pattern of response varied between species. These results define operational staining conditions for the assay and describe the performance of the complete diluent formulations under the conditions tested.

The selected incubation times were the shortest among those tested that satisfied the predefined criterion of five evaluable fields in every biological replicate. Because the three fluorochromes were added sequentially, these settings corresponded to total staining periods of 60 min for *A. mellifera* and 90 min for *S. mexicana*. This is a practical difference for applying the staining protocol; the experiment was not designed to determine its physiological cause. Staining was performed at 22–25 °C, and semen was obtained by different procedures in the two species. These conditions should be considered when interpreting the between-species comparison.

The individual vesicle-derived sperm suspensions of *S. mexicana* were maintained and evaluated separately, without pooling. Microscopic fields and individual spermatozoa were subsamples within each suspension.

Sperm vitality was assessed by simultaneous evaluation of Hoechst 33342 and propidium iodide (PI) fluorescence. Spermatozoa exhibiting blue nuclear fluorescence without PI staining (Hoechst-positive/PI-negative) were classified as live, whereas PI-positive spermatozoa were classified as dead according to this staining assay. Sperm vitality was defined as the percentage of spermatozoa classified as live among all spermatozoa evaluated. This live–dead classification reflected plasma membrane integrity and was not considered a measure of overall sperm function or fertilizing capacity.

FITC-PNA provided an additional, localized fluorescence pattern that was evaluated separately. Spermatozoa exhibiting localized green fluorescence in the presumed acrosomal region were classified as having damaged acrosomes, whereas those without detectable green fluorescence in this region were classified as having intact acrosomes according to the operational criterion used in this study. In *A. mellifera*, an average acrosome length of 4.02 µm and a total sperm head length of 8.74 µm have been reported [17]. These dimensions provide a morphological reference for interpreting localized fluorescence in the anterior head region. However, correspondence in size and position alone does not validate the anatomical specificity of FITC-PNA staining. This operational classification was not interpreted as evidence of a physiological acrosome reaction. The experiment did not determine whether the observed pattern resulted from collection, sample handling, storage, or staining. Its anatomical specificity and functional significance require further validation. These dimensions provide a morphological reference for interpreting localized fluorescence in the anterior head region. However, correspondence in size and position alone does not validate the anatomical specificity of FITC-PNA staining. The observed FITC-PNA pattern was classified as acrosomal damage according to the operational criterion used in this study; it was not interpreted as evidence of a physiological acrosome reaction. The experiment did not determine whether this pattern resulted from collection, sample handling, storage, or staining. Its anatomical specificity and functional significance require further validation.

In *A. mellifera*, modified Kiev yielded the highest estimated sperm vitality after one week of storage, although it did not differ significantly from FEM7. Previous studies have investigated Kiev-based extenders for honey bee sperm cryopreservation [18] and the effects of diluent and incubation conditions on sperm motility [14]. These studies provide context for considering the physicochemical environment during semen handling. However, the present experiment compared complete formulations and did not isolate the effects of buffering agents, energy substrates, bovine serum albumin, or individual ions. Their contributions to the observed extender performance therefore remain hypotheses rather than demonstrated mechanisms.

The observed differences among complete extender formulations may reflect their physicochemical properties, but the contribution of individual components and their effects on sperm function were not determined in this study. Although detailed physiological information on *S. mexicana* sperm remains limited, species-specific differences in sperm structure and ultrastructure have been documented among Meliponini [19]. One hypothesis is that differences in membrane permeability or tolerance to extracellular osmotic conditions modify the response of *S. mexicana* spermatozoa to the evaluated formulations. Differences in solute permeability could alter the magnitude and duration of cell-volume changes during dilution. However, the ultrastructural evidence does not establish these membrane properties, and their contribution to the observed results remains to be tested. Osmotic stress and plasma membrane alterations have been discussed in the broader sperm-preservation literature [20], providing a basis for investigating these mechanisms in stingless bees.

The contrasting responses observed between *A. mellifera* and *S. mexicana* must be interpreted in the context of the different collection procedures. Because species and collection method were not evaluated independently, their contributions to the observed differences cannot be separated. These results therefore describe extender performance under the conditions tested and cannot be attributed exclusively to intrinsic differences between species. Nevertheless, the importance of sperm storage and subsequent use in honey bee reproduction [21] provides a biological rationale for evaluating semen extenders. In the present study, this evaluation was limited to fluorescence-based indicators under the collection and storage conditions tested.

Although the present study did not evaluate cryopreservation directly, the favorable performance of FEM7 in *S. mexicana* under the conditions tested identifies this extender as a candidate for future cryopreservation studies in stingless bees. A sperm cryopreservation protocol has recently been reported for *S. bipunctata* using two extender formulations [22], providing a methodological precedent for semen preservation in *Scaptotrigona*. However, its applicability to *S. mexicana* remains to be evaluated. FEM7 has previously shown favorable results in honey bee sperm preservation [15]. Its performance in the present study may reflect a physicochemical environment favorable to sperm membrane integrity, although reduced osmotic stress and the contributions of individual components, including carbohydrates, were not directly assessed. Future studies should evaluate the compatibility of FEM7 with different cryoprotectants and freezing protocols to determine its suitability for long-term germplasm conservation.

SS consistently resulted in the lowest sperm vitality in both *A. mellifera* and *S. mexicana*, indicating that sperm maintenance requires an appropriate physicochemical environment rather than the presence of individual ions alone. In honey bees, sperm motility and survival are influenced by diluent composition, including its ionic and osmotic properties [23,24]. The spermathecal fluid of *A. mellifera* queens contains relatively high concentrations of Na^+^ and K^+^ [25], and this hypertonic ionic environment has been proposed to reversibly suppress sperm motility and respiration, thereby contributing to long-term sperm survival [26]. Therefore, the reduced sperm vitality observed in SS may be associated with its limited ionic composition and the absence of other components required to maintain a suitable extracellular environment.

Acrosomal damage was detected exclusively in spermatozoa classified as dead by PI staining. In these cells, localized green FITC-PNA fluorescence in the acrosomal region indicated damage according to the classification criterion used in this study. The combined use of Hoechst 33342, PI, and FITC-PNA allowed sperm vitality and acrosomal integrity to be assessed separately in the same sample. Because no washing steps were performed, unbound fluorescent reagents remained in the suspension and could contribute to background fluorescence. Classification was based on the cellular fluorescence patterns defined in Section 2.5. Background fluorescence was not quantified separately, so its contribution to image interpretation was not assessed quantitatively.

Despite the significant differences observed among extenders, several limitations should be considered when interpreting these results. Semen collection differed between species, with ejaculated semen obtained from *A. mellifera* and sperm recovered directly from the seminal vesicles in *S. mexicana*. These differences may influence sperm composition and, consequently, the response to different extenders. The diluent experiment was performed in vitro after one week of storage at 22 °C. This storage period did not apply to the incubation-time experiment. The diluent results therefore describe sperm quality at a single storage endpoint. Future studies should therefore examine multiple storage intervals to determine the temporal stability of sperm vitality and acrosomal integrity under different preservation conditions. Future studies should also evaluate the relationship between the fluorescence-based indicators described here and functional reproductive parameters such as sperm storage efficiency, fertilization success, and long-term survival after artificial insemination or cryopreservation.

Overall, this study demonstrates that both fluorescence staining conditions and semen extender composition must be optimized independently for each bee species. The standardized protocol developed here provides a reliable method for simultaneously evaluating sperm vitality and acrosomal integrity in *A. mellifera* and *S. mexicana*. Moreover, the contrasting responses observed among extenders indicate that preservation protocols developed for honey bees cannot be directly extrapolated to stingless bees without prior validation. The favorable performance of FEM7 in *S. mexicana* also identifies this extender as a promising candidate for future studies on semen preservation and cryopreservation in Meliponini, thereby contributing to the development of assisted reproductive technologies and genetic resource conservation programs for managed and native bees.

## 5. Conclusions

Among the incubation times tested, the shortest times yielding consistently evaluable fluorescence were 20 min per fluorochrome for *A. mellifera* and 30 min per fluorochrome for *S. mexicana*. Sperm vitality, defined here by Hoechst 33342/PI live–dead classification, differed among the evaluated diluents after one week of storage. Modified Kiev yielded the highest estimated value in *A. mellifera*, without a significant difference from FEM7, whereas FEM7 yielded the highest estimated value in *S. mexicana*. These findings support the use of explicitly defined staining conditions and species-aware evaluation of complete diluent formulations. Further work is required to establish the anatomical and functional meaning of FITC-PNA staining and to test whether the observed laboratory outcomes predict reproductive performance.

## Data Availability

Data are contained within the article.

## References

[1] Aizen M.A., Garibaldi L.A., Cunningham S.A., Klein A.M. (2009). How much does agriculture depend on pollinators? Lessons from long-term trends in crop production. Ann. Bot..

[2] Potts S.G., Biesmeijer J.C., Kremen C., Neumann P., Schweiger O., Kunin W.E. (2010). Global pollinator declines: Trends, impacts and drivers. Trends Ecol. Evol..

[3] Baer B., Collins J., Maalaps K., Den Boer S.P.A. (2016). Sperm use economy of honeybee (*Apis mellifera*) queens. Ecol. Evol..

[4] Al-Lawati H., Kamp G., Bienefeld K. (2009). Characteristics of the spermathecal contents of old and young honeybee queens. J. Insect Physiol..

[5] Rangel J., Shepherd T.F., Gonzalez A.N., Hillhouse A., Konganti K., Ing N.H. (2021). Transcriptomic analysis of the honey bee (*Apis mellifera*) queen spermathecae reveals genes that may be involved in sperm storage after mating. PLoS ONE.

[6] Collins A.M., Donoghue A.M. (1999). Viability assessment of honey bee, *Apis mellifera*, sperm using dual fluorescent staining. Theriogenology.

[7] Pettis J.S., Rice N., Joselow K., VanEngelsdorp D., Chaimanee V. (2016). Colony Failure Linked to Low Sperm Viability in Honey Bee (*Apis mellifera*) Queens and an Exploration of Potential Causative Factors. PLoS ONE.

[8] Yániz J.L., Silvestre M.A., Santolaria P. (2020). Sperm Quality Assessment in Honey Bee Drones. Biology.

[9] Real-Luna N., Rivera-Hernández J.E., Alcántara-Salinas G., Rojas-Malavasi G., Morales-Vargas A.P., Pérez-Sato J.A. (2022). Las abejas sin aguijón (Tribu Meliponini) en los agroecosistemas de América Latina. Rev. Mex. Cienc. Agríc..

[10] Meneses H.M., Koffler S., Freitas B.M., Imperatriz-Fonseca V.L., Jaffé R. (2014). Assessing Sperm Quality in Stingless Bees. Sociobiology.

[11] Locke S.J., Peng Y., Cross N.L. (1990). A supravital staining technique for honey bee spermatozoa. Physiol. Entomol..

[12] Egyptien S., Dewals B., Ectors F., Brutinel F., Ponthier J., Deleuze S. (2023). Validation of Calcein Violet as a New Marker of Semen Membrane Integrity in Domestic Animals. Animals.

[13] Almadaly E., El-Kon I., Heleil B., Fattouh E.-S., Mukoujima K., Ueda T., Hoshino Y., Takasu M., Murase T. (2012). Methodological factors affecting the results of staining frozen–thawed fertile and subfertile Japanese Black bull spermatozoa for acrosomal status. Anim. Reprod. Sci..

[14] Yániz J., Palacín I., Santolaria P. (2019). Effect of chamber characteristics, incubation, and diluent on motility of honey bee (*Apis mellifera*) drone sperm. Apidologie.

[15] Rajamohan A., Danka R.G., Hopkins B.K., Rinehart J.P. (2020). A non-activating diluent to prolong in vitro viability of *Apis mellifera* spermatozoa: Effects on cryopreservation and on egg fertilization. Cryobiology.

[16] Dulbecco R., Vogt M. (1954). Plaque formation and isolation of pure lines with poliomyelitis viruses. J. Exp. Med..

[17] Bratu I.C., Igna V., Simiz E., Dunea I.B., Pătruică S. (2022). The Influence of Body Weight on Semen Parameters in *Apis mellifera* Drones. Insects.

[18] Hopkins B.K., Herr C. (2010). Factors affecting the successful cryopreservation of honey bee (*Apis mellifera*) spermatozoa. Apidologie.

[19] Zama U., Lino-Neto J., Dolder H. (2004). Structure and ultrastructure of spermatozoa in Meliponini (stingless bees) (Hymenoptera: Apidae). Tissue Cell.

[20] Watson P.F. (2000). The causes of reduced fertility with cryopreserved semen. Anim. Reprod. Sci..

[21] Baer B. (2005). Sexual selection in Apis bees. Apidologie.

[22] Marques L.S., Blochtein B., Dos Santos C.F., De Freitas T.R., Dos Santos Teixeira N., De Souza França T., Rodrigues R.B., Dantas R.V., Pedro D.S. (2026). A practical and feasible protocol for cryopreservation of stingless bee sperm. Apidologie.

[23] Lensky Y., Schindler H. (1967). Motility and reversible inactivation of honeybee spermatozoa in vivo and in vitro. Ann. De l’Abeille.

[24] Verma L.R. (1978). Biology of honeybee (*Apis mellifera* L.) spermatozoa. 1. Effect of different diluents on motility and survival. Apidologie.

[25] Gessner B., Gessner K. (1976). Inorganic ions in spermathecal fluid and their transport across the spermathecal membrane of the queen bee, *Apis mellifera*. J. Insect Physiol..

[26] Verma L.R. (1973). An ionic basis for a possible mechanism of sperm survival in the spermatheca of the queen honey bee (*Apis mellifera* L.). Comp. Biochem. Physiol. Part A Physiol..

